# Leveraging Feedback From Families of Children With Autism to Create Digital Support for Service Navigation: Descriptive Study

**DOI:** 10.2196/56043

**Published:** 2024-08-14

**Authors:** Meghan Burke, Chak Li, Waifong Catherine Cheung, Adriana Kaori Terol, Amanda Johnston, Stephen M Schueller

**Affiliations:** 1 Department of Special Education, Vanderbilt University Nashville, TN United States; 2 Northern Illinois University Dekalb, IL United States; 3 University of Illinois at Urbana-Champaign Champaign, IL United States; 4 University of California at Irvine Irvine, CA United States

**Keywords:** human-centered design, autism, service access, families, digital support, autistic children, autistic, children, child, app, apps, application, applications, digital tool, tool, tools

## Abstract

**Background:**

It is difficult for families to navigate and access services for their children with autism. Barriers to service access are compounded among families from low-resourced backgrounds.

**Objective:**

The purpose of our study was to explore the development of an app to facilitate access to services among families of children with autism from low-resourced backgrounds. Our specific aims were to explore feedback from an advisory board about the app and to explore feedback from navigators about the app.

**Methods:**

Via a multistage codevelopment process, we elicited feedback from 5 key parties: the research team, a community organization, the app development team, the advisory board, and family navigators. Collectively, 36 individuals provided feedback about the development of the app via individual interviews, focus groups, observations, and surveys. The key features of the app included a dashboard showing the service needs of the family and related resources, a messaging feature between the family, the navigator, and the supervisor, and a fidelity checklist and evaluation feature.

**Results:**

The advisory board provided feedback about the app to increase its user-friendliness, include the ability to develop an action plan, improve the identification of needed services, and add information about service providers. Navigators suggested that the app should connect navigators to one another, have a clearer purpose for the notes section, and reflect an easier log-in process. Navigators also wanted training to role-play using the app. After participating in a role play using the app, navigators reported significantly more satisfaction with the app and greater usefulness (*P*<.001).

**Conclusions:**

Our work sheds light on the importance of eliciting feedback from end users, especially users who are often overlooked by the research community and app developers. Further, it is important to elicit feedback in multiple ways to improve the app.

## Introduction

It is difficult for families to navigate service delivery systems; it can be especially challenging for families to access services for their children with autism. Autism services are fragmented as they are provided by multiple systems, including schools, private providers, hospitals, and clinics. The lack of a unified system of care puts the onus on parents to act like case managers—identifying, locating, and accessing services [1-3]. However, service navigation is challenging, and parents often struggle to: identify the appropriate mix of services for their child; find resources to obtain services; communicate with providers; and maintain records of correspondence with multiple service systems [4,5]. Although families report many barriers to accessing services (eg, lack of transportation, insurance denials, co-pays, and provider shortages [6]), lack of knowledge about services is one of the most common and most malleable barriers [7].

Digital support can be helpful to increase access to and knowledge of services. In populations other than families of children with autism (eg, adults with mental health diagnoses and other types of disabilities), digital supports have demonstrated success to help individuals access services [8]. For example, incorporating measures of mental health into electronic health record systems has resulted in greater efficiency in accessing mental health services [9]. With respect to autism, many digital mental health interventions have been developed, addressing various areas including executive function, language, emotional needs, and time management [10]. Although interventions have been impactful in those target areas, they still need to be introduced to children with autism, either through existing or new delivery pathways. Given that digital supports have yet to address access to services among children with autism and the challenges often involved in developing and implementing new technologies, a focus on repurposing technologies to support access to existing care rather than creating new interventions could be a fruitful area of development. Such approaches could also leverage the affordances of technologies to connect people and organize information rather than delivering complex evidence-based practices through digital means.

In particular, families from low-resourced backgrounds are especially overlooked in autism care, research, and innovation. “Low-resourced” refers to individuals with low income or one of the following: the primary caregiver has a high school diploma or less; the primary caregiver is unemployed; and the family receives governmental assistance [11]. Families from low-resourced backgrounds often face great economic and resource demands, which, in part, occlude access to services [12,13]. For example, families of children with autism who are low-income are significantly less likely than high-income families to: receive care from a specialist; have a personal doctor; receive timely, acute care; and visit a doctor for preventive care [14]. Also, families of children with autism who are low-income are almost 3 times more likely than high-income families to struggle to obtain advice about services [15]. Parents with a high school diploma or less (vs college education) are 2-4 times less likely to access certain medical, therapeutic, and educational services for their offspring with autism [16]. Further, Latinx (vs White) children with autism from low-resourced backgrounds are at particular risk for service disparities, which are compounded among monolingual, Spanish-speaking families [17]. Despite the rhetoric of technologies opening access to traditionally underserved and marginalized groups [18], low-resourced populations are often the last to receive innovations; indeed, few digital supports address the needs of families from low-resourced backgrounds.

Co-design and codevelopment, that is, involving populations in the design of the technology from the start through structured methods, has been proposed as one solution to address this gap [19]. At the core of co-design is the idea that the populations that will be impacted by the technologies are best positioned to articulate how such technologies can meet their needs and incorporate their interests. Although a range of studies have used co-design processes in the service of developing digital mental health interventions [20], these studies have had considerable variation in the stage of involvement, co-design methods, and invested parties included. As such, several models have been developed to consider the contributions of co-design processes to mental health intervention development, including the accelerated creation-to-sustainment (ACTS) model [20] and the discover, design, build, and test (DDBT) framework [21]. Consistent across both ACTS and DDBT is the notion that implementation of the intervention needs to be considered alongside the design of the intervention itself. Therefore, both models apply co-design methods to the development of the implementation strategy and interventions. These models differ in that DDBT is applied more broadly to consider digital and non-digital mental health interventions and apply concepts from human-centered design to psychosocial interventions broadly. The ACTS model is specifically focused on technology-enabled services, based on the recognition that digital mental health interventions include both technological and service components [20]. Both models can contribute important lessons and structure to apply co-design methods when designing digital supports for mental health services.

To this end, the purpose of this study was to explore the development of an app to facilitate families of children with autism from low-resourced backgrounds to access services. Specifically, the aims of this study were to explore the feedback from an advisory board about the app and explore the feedback from navigators about the app. The app was conceived and tested with the goal of implementability (ie, able to be deployed and used by families seeking care and individuals supporting care). Often, input from end users is not considered when designing a technological tool [22]; most technologies are developed in controlled trials, rather than the deployment setting, thus impacting implementation [23,24]. We addressed both gaps by including families of children with autism from low-resourced backgrounds during the development of the app. In future research, our intention is for service navigators (who are parents of older children with autism) to use the app to guide the families of young children with autism to access services. In this study, we describe the development process, including its focus on human-centered design by partnering with community organizations and families. This study may be helpful to others in developing successful digital interventions for families of youth with autism.

## Methods

### Design

We engaged in a multistage codevelopment process, soliciting input from 5 key invested parties at various stages. The parties included the research team, a community organization (the Parent Training and Information Center [PTI]), the app development team, the advisory board, and family navigators. Our co-development process was based on several models (ie, the ACTS model [20] and the DDBT framework [21]) that outline co-design activities and discuss opportunities to combine co-design and implementation science methods. At different stages, we collected either formal (eg, established survey measures) or informal (eg, observations of feedback during meetings) data, including feedback for formative purposes of iterating the app and for summative purposes of evaluating the resultant project. As such, we have descriptive information about our process, qualitative data from interviews and focus groups, and quantitative data from our summative evaluation. Below, we describe the ethical considerations, participants in the study, the procedures (ie, stages of app development and testing), and the data analysis.

### Ethical Considerations

Vanderbilt University’s institutional review board's approval was received for this study (approval number 231178). All participants provided informed consent to participate in this study. All data were deidentified for data analysis. Participants were compensated for their participation. Specifically, each advisory board was compensated with a US $30 gift card. Each navigator was compensated a US $100 gift card.

### Participants

Across the 5 key parties, there were 36 people. Formal data were only collected from the advisory board members and family navigators as their backgrounds and characteristics aligned with the app’s end users (eg, families of children with autism from low-resourced backgrounds).

#### Advisory Board Participants

To be included in the advisory board, participants needed to have a child with autism, speak English or Spanish, and self-identify as low-resourced (ie, identify as having low income or one of the following: the primary caregiver has a high school diploma or less; the primary caregiver is unemployed; and the family receives governmental assistance [11]). Recruitment methods included word-of-mouth to the autism community as well as sharing information about the study via social media, flyers, and websites. The research team partnered with a PTI, a federally funded agency designed to educate and empower parents of children with disabilities, to recruit participants for the advisory board. There is at least one PTI in every state of the country. Altogether, the advisory board consisted of 8 mothers of children with autism from low-resourced backgrounds. Recruitment ended when a redundancy of themes was reached. Each participant completed a consent form before participating in the data collection.

#### Family Navigator Participants

The second group of participants was comprised of 19 family navigators. To be included in this study, individuals needed to: have a child with autism who was older than 5 years and receive an autism diagnosis from a health care provider, participate in the family navigator training, and self-identify as low-resourced [11]. Participation was limited to parents of children who were 5 and older to ensure that the participants had sufficient experience in navigating service delivery systems to, ultimately, serve as navigators for families of 3- to 5-year-olds on the spectrum. To determine the autism diagnosis, all participants met the cutoffs using the Social Communication Questionnaire [25], which was delivered by the study coordinator after the participant provided informed consent to participate in the study. The navigators completed 12 two-hour training sessions to learn how to support families of children with autism in accessing services using the app. Recruitment methods included word-of-mouth in the autism community as well as sharing information about the study via social media, flyers, and websites; the PTI also assisted with recruiting participants. The PTI staff were also parents of individuals with autism themselves. See Table 1 for participant characteristics.

**Table 1:** Participant (advisory board and navigator) demographics.

Characteristic		Advisory board members (n=8)			Navigators (n=19)		
**Gender, % (n)**							
	Female	100 (8)			95 (18)		
**Race, % (n)**							
	White	12 (1)			37 (7)		
	Hispanic or Latinx	63 (5)			37 (7)		
	Black or African American	25 (2)			16 (3)		
	Asian	—			5 (1)		
	More than one race	—			5 (1)		
**Marital status, % (n)**							
	Married	63 (5)			68 (13)		
**Annual household income (US $), % (n)**							
	≤15,000	—			5 (1)		
	15,000-29,999	12 (1)			16 (3)		
	30,000-49,999	12 (1)			26 (5)		
	50,000-69,000	37 (3)			21 (4)		
	70,000-99,999	12 (1)			5 (1)		
	≥100,000	12 (1)			21 (4)		
	Missing	12 (1)			5 (1)		
**Educational background, % (n)**							
	High school diploma			63 (5)			—
	Some college			—			37 (7)
	4-Year degree			25 (2)			37 (7)
	Graduate or professional degree			12 (1)			26 (5)
**Employment, % (n)**							
	Currently employed		50 (4)			31.58 (6)	
**Governmental assistance, % (n)**							
	Receives governmental assistance	75 (6)			36.84 (7)		

### Procedures

Interested individuals contacted the research team to participate and be on the advisory board or as a family navigator. A researcher conducted a screening to ensure the individual met the inclusionary criteria. If the individual met the inclusionary criteria, the researcher sent the consent form to the participant.

**Figure 1:**
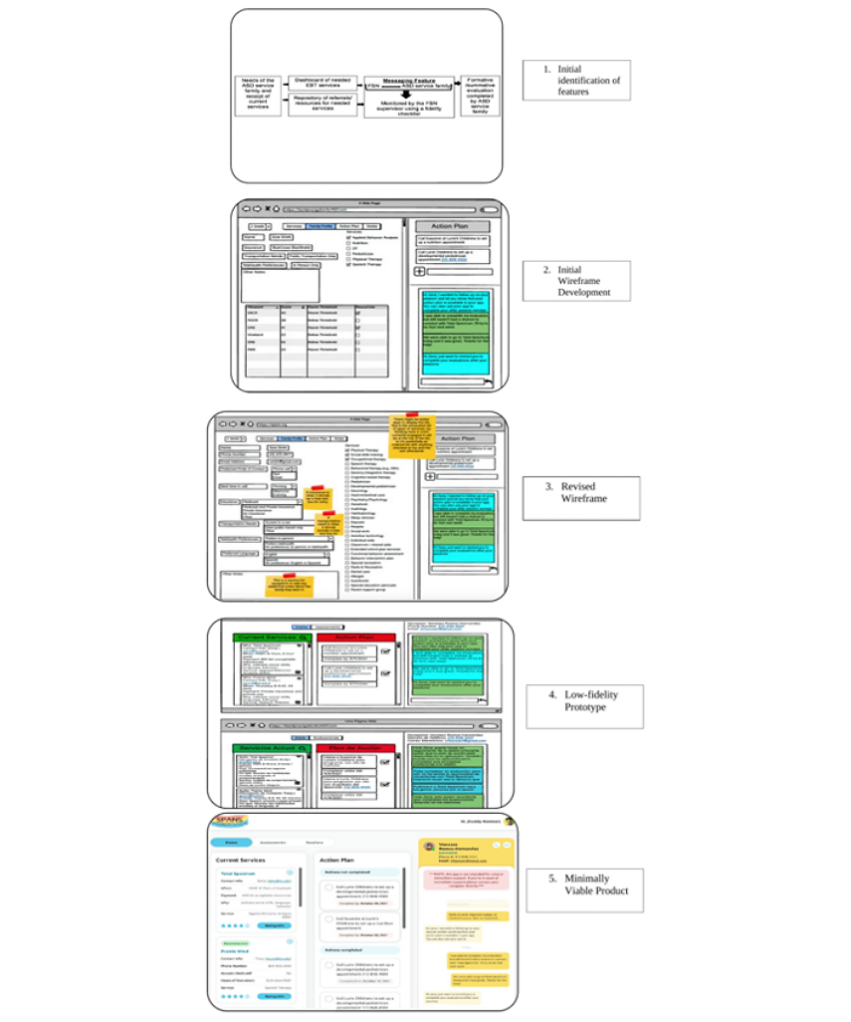
Five stages of the app development process.

The app development process followed a five-step sequence: (1) initial identification of features between the research team and the PTI; (2) development of the initial wireframe between the research team and the app development team; (3) revised wireframe; (4) low-fidelity prototype of the app; and (5) a minimally viable product to be informed by the advisory board and navigators. The team meetings are described later in the manuscript. See Figure 1 for the overall process.

#### Initial Identification of Features and Wireframes Resulting in a Prototype

Initially, when proposing this project, the research team and the PTI identified the main features for the app. These features were derived from their lived experiences as parents of children with autism as well as their collective 50 years of experience educating and empowering families of children with autism from low-resourced backgrounds. The features were combined into a figure to summarize the main purpose of the app. The features included: a dashboard showing the service needs of the family of a young child with autism and related resources; a messaging feature to facilitate and document communication between the family navigator supervisor, family navigator, and family of a young child with autism; and a fidelity checklist and evaluation feature to measure implementation outcomes. We shared the figure with the app developer and together developed the initial wireframe mock-up of the app (Multimedia Appendix 1).

#### Individual Feedback From the Advisory Board to Inform the App

The wireframe mock-up was reviewed by the advisory board. Specifically, individual interviews were conducted with each of the advisory board members. On average, interviews lasted 60 minutes. Each interview was transcribed verbatim. We revised the wireframe mock-up based on their feedback. See Multimedia Appendix 2 for the mock-up of the wireframes with post-its showing suggested changes.

#### Group Feedback From the Advisory Board to Inform the App

We shared the revised wireframe mock-up with the advisory board at a large group, 1-hour meeting held over Zoom. Advisory board members provided collective feedback about the revised wireframes. Specifically, prior to the meeting, the advisory board received the revised wireframes for review. During the meeting, the research team gave an overview of the wireframes, elicited feedback via polls from the advisory board about the revised wireframes, and then held an open discussion about the app features and next steps. For the polls, advisory board members provided feedback on the importance of each feature (eg, the communication feature allowing the navigator, supervisor, and family to dialogue with one another; the referral services feature; the notification feature; the assessment feature; the evaluation feature; and the action plan). After the polls, there was an unstructured discussion wherein the advisory board members could provide additional feedback about the app. The meeting was transcribed verbatim. We then revised the wireframe mock-up again based on the feedback and developed the app (Multimedia Appendix 3).

#### Navigator Feedback During the Training

Family navigators completed 12 two-hour training sessions (24 total hours of training), which were recorded and transcribed. Following the final training session, navigators completed an electronic presurvey about the app. At that point, navigators requested an additional session solely focused on the app and how to use it with families (eg, role play). An additional session was created in response to the navigators’ feedback. In this session, navigators practiced using the app with research team members who role played as families of children with autism. After the session, the navigators again completed the same electronic survey about the app (ie, the postsurvey).

The survey included several measures: (1) the App Satisfaction Questionnaire [26] consisted of 3 items asking respondents about their overall satisfaction with the app. Items included: “Overall, I am satisfied with the ease of using the app”; “Overall, I am satisfied with the amount of time it takes to use the app”; and “Overall, I am satisfied with the support information (instructions, examples) to use the app.” Items were scored using a 7-point Likert-type scale, where 1=*strongly disagree* and 7=*strongly agree*. Higher scores indicate higher levels of positive experiences; (2) the System Usability Scale [27] consisted of 10 items asking respondents their perceptions of the usability of the app. Example items included: “I think that I would like to use the app frequently” and “I found the app unnecessarily complex.” Items were scored using a 5-point Likert-type scale, where 1=*strongly disagree* and 5=*strongly agree*. After reverse-coding specific items, higher scores indicate higher levels of positive experiences; and (3) the Usefulness, Satisfaction, and Ease of Use ([28]) consisted of 30 items asking respondents their ability to use the functions of the app. Example items included: “The app will help me be more productive when working with families” and “The app could give me more control over providing navigation support to families.” Items were scored using a 7-point Likert-type scale, wherein 1=*strongly disagree* and 7=*strongly agree*. Higher scores indicate more positive experiences (Multimedia Appendix 4).

### Analyses

All qualitative data (eg, the individual interviews, comments made during the advisory board meeting, and the navigator training) were transcribed verbatim. The research team read the transcripts multiple times to familiarize themselves with the data [29]. Team members independently coded the data using thematic analysis and open-axial coding to identify key themes regarding the development and revision of the app’s content, functionality, and design. Specifically, using a line-by-line approach, the team members individually coded all the data. Each piece of data was compared with the other data, highlighted, and annotated with a specific phrase [30]. Each new piece of data was compared with previously coded data to check if the new data reflect a new code or was an existing code. The team came together to compare codes and resolve differences. They grouped the codes into categories and clustered the categories into themes grounded in the data. Notably, each research team member had experience collecting and analyzing qualitative data.

All quantitative data (eg, survey data) were input into a statistical program. The research team conducted descriptive statistics (eg, means, SDs, and ranges) to characterize the data. To examine pre-post differences, paired *t* tests (2-tailed) were conducted with Cohen *d* as the effect size.

## Results

### Individual Feedback From the Advisory Board

The feedback from the advisory board interviews was organized into four themes: (1) the need for a user-friendly design, (2) the ability to develop an action plan, (3) the identification of needed services, and (4) information about service providers. Regarding user-friendly design, advisory board members wanted the app to be accessible for all families—including families who were not familiar with technology. Several advisory board members reported that families from low-resourced backgrounds may not be familiar with technology. Thus, having a user-friendly design was important. To this end, advisory board members had several suggestions: using simple language, having visuals (eg, familiar icons), and being concise when presenting information. For example, a participant mentioned, “It [The app] should be very clear for families, like if I want to contact someone, I should press this icon. This icon is to access the calendar and see my planned or due activities. Here are my notes or reminders of things I have to ask providers or navigators.” We carefully reviewed this feedback and, whenever possible, focused on accessibility in the app (eg, simplifying the language and reducing extraneous text). Second, advisory board members wanted the app to include an “action plan” wherein they could identify steps to secure services. Participants reported that the app already included the current and needed services of the family. The action plan would help families identify the discrete steps needed to access needed services. For example, advisory board members reported that the action plan could help families contact service providers and schedule services. Thus, we emphasized the action plan in the app by making it a featured tab as well as adding time stamps for when an action should be completed. Finally, advisory board members had 2 suggestions related to the service providers in the app: a clear description of each service provider and a filter option for each service provider. Regarding the former, advisory board members requested that the app include a description of each service provider, with a participant stating that, “It would be good to add the link for the website of the service provider so that the app redirects you to their website.” To this end, the app was revised to include links to each service provider’s website in addition to their phone number and email addresses. Regarding the latter, advisory board members requested that each service have a filter. Specifically, multiple members requested that the app detail the types of insurance covered by each service provider because, as an advisory board member reported, “[Families] may still need to look for do they accept certain kind of insurance” for the identified services. This suggestion was addressed accordingly and also added as a feature of the app. See Textbox 1 for more quotes representing each theme.

**Themes and representative quotes identified from individual advisory board member interviews.**

User-friendliness and design

“But on a phone, I think that it's more of a, you know, you have like your one screen but at the bottom you might have your buttons, or maybe you might have it on the side.”

“When it comes to families and you are targeting families who may not have the resource, that means they may not have literacy levels and all of that. Then we have to simplify it [the language].”

“It should be very clear for families, like if I want to contact someone, I should press this icon, this icon is to access the calendar and see my planned or due activities, here are my notes or reminders of things I have to ask providers or navigators”

“It may be helpful to provide a little bit of training about what is an app. There are some parents, maybe those who are older or who come from other countries may not be familiarized with technology...”

#### Group Feedback From the Advisory Board

During the web-based advisory board meeting, feedback was solicited in relation to the strengths of the app and its needs for improvement. Advisory board members were asked about the most important features of the app (ie, the communication feature allowing the navigator, supervisor, and family to dialogue with one another; the referral services feature; the notification feature; the assessment feature; the evaluation feature; and the action plan). Their responses were unanimous, finding that communication was the most important feature of the app. Specifically, advisory board members reported that it was most important that the app enabled navigators to dialogue with families (and vice versa). For example, a participant stated, “It [communication] is important because it assists in building trust between families and navigators and allows families to learn more.” Also, another participant mentioned, “[Communication features provide] opportunities for families to ask questions of one another to assist in the peer-to-peer network.” We ensured that the app included 3 types of communication: the ability for the navigator to communicate with the supervisor; the ability of the navigator to communicate with the family; and the ability of the navigator to write a note to themselves.

Regarding a feature in need of improvement, members reported that the evaluation feature was the least useful in the app. The evaluation feature focused on offering an option for families to evaluate their experiences with navigators. Advisory board members reported that families should have the opportunity to provide feedback about their navigators, but some members reported that families may feel uncomfortable providing feedback in the app. To this end, some members suggested providing a different medium for families to provide feedback. For example, instead of using the app, some members suggested that families should provide verbal feedback to navigators. Alternatively, other members suggested that the app was an acceptable mechanism to provide feedback, but the feedback form should be simple. For example, a member suggested that the family should provide a number of stars to rate their navigators instead of responding to specific questions. The research team and the app developer discussed this potential change, but, due to the research nature of this project, we retained the questions for evaluation data. To partially accommodate this request, we added the ability to rate the navigator (using stars) in addition to the original evaluation questions (ie, “How useful was this session?”; “How would you rate your experience with your navigator?”; and “Do you have any confidential comments only for your navigator’s supervisor?”).

#### Navigator Feedback During the Training

Throughout the navigator training, navigator feedback fell under four main themes: (1) offer a way for navigators to connect to one another; (2) clarify the nature of the notes section in the app; (3) improve the ease of logging into the app; and (4) offer training to role play with the app. First, navigators wanted the app to offer a way for navigators to connect to one another. Specifically, they suggested that the app include a message board, portal, or messaging service to allow the navigators to speak to one another, share resources, and troubleshoot. A navigator stated, “Maybe there is a group page… so we can build our knowledge base about services...and share scenarios where we are stuck and rely on each other.” Unfortunately, we did not have sufficient funding to accommodate this request. However, we plan to collect data from the navigators to determine if they create a workaround (eg, create their own Facebook group so they can communicate with one another) to address this issue. Second, navigators asked questions about the notes section in the app. Specifically, navigators wanted to ensure that the notes that they sent to the supervisor could not be seen by the family. This change was made in the app. Third, some navigators struggled with logging into the app; the navigators who struggled to log into the app questioned whether there would be ongoing technical assistance for the navigators about the app. The team made sure that there was a team member available to respond to all inquiries about the app from the navigators and families. Finally, navigators requested having a separate training session wherein they could role play using the app. Specifically, navigators wanted to watch an example role play with the app, between a navigator and a family. A navigator reported, “I would like to do a true role play wherein we watch a first meeting between the navigator and the family.” Accordingly, the booster session included navigators watching a role play of the interactions between a navigator and a family, followed by participating in a role play themselves. See Multimedia Appendix 5 for the final product.

#### Navigator Survey Feedback

At the end of the navigator training, navigators reported moderate levels of satisfaction (mean 4.21, SD 7), usability (mean 3.07, SD 5), and ease of use for the app (mean 4.62, SD 7). Although these values are above the midpoint of the scale, they were in the “neither agree nor disagree” to “somewhat agree” range in terms of the 7-point Likert scale items. Notably, the abovementioned qualitative data suggested that their comfort with the app could improve. After the additional session, scores on all measures improved. Specifically, with respect to the App Satisfaction Questionnaire and the Usefulness, Satisfaction, and Ease of Use measures, scores improved significantly after the session (*P*<.001), with large effect sizes ranging from 1.25 to 2.07 (Table 2).

**Table 2:** Descriptive statistics of navigator survey feedback.

Variable	Pre-booster, mean (SD)	Pre-booster, α	Post-booster, mean (SD)	Post-booster, α	*t*	*P*	Cohen *d*
App Satisfaction Questionnaire	4.21 (1.00)	.96	5.64 (1.03)	.94	7.46	<.001	2.07
System Usability Scale	3.07 (.18)	.86	3.31 (.48)	.88	1.99	.06	0.55
Usefulness, Satisfaction, and Ease of Use	4.62 (.68)	.95	5.48 (.86)	.98	4.50	<.001	1.25

### Final Version of the Digital Support

The aforementioned steps and data sources are reflected in the list of our key learnings from the project. See Multimedia Appendix 6 for the list. Researchers interested in a checklist of co-design activities may consider the checklist provided in a review conducted by Bevan Jones et al [20] about co-designed digital mental health technologies. The final version of the app is available in Multimedia Appendix 7. Specifically, upon logging into the app, the user sees the profile of a family, including their preferred mode of contact, current services, recommended services, action plan, and a chat feature to communicate with the navigator. To identify relevant service providers, the family and navigator can input their preferred parameters into the “Filter Services” feature. There, they can indicate the type of insurance the provider should accept, whether telehealth is an option, whether the provider speaks Spanish, and the geographic location of the provider. Upon entering the parameters, the app will generate a list of providers that meet the parameters for each recommended service. The app also includes an action plan that details the next steps for the family to seek the recommended service providers. The app includes a Notes feature, allowing the family and navigator to write notes about their contact with one another. At the end of each meeting with a navigator, the family can provide formative feedback about their experience with the navigator.

## Discussion

In this study, we used human-centered design methods [31] and incorporated the input of families of children with autism from low-resourced backgrounds in the development of an app to facilitate access to services. The aim of this formative research was to better understand the feedback from various invested parties and contribute to the development of that app. Thus, a key finding was the importance of offering different ways for diverse groups of people to provide feedback during the development process. In our study, each party (eg, advisory board member, PTI, navigators) and method of involvement (eg, participating on an advisory board, comments during a training, role play) yielded unique and important feedback. The synthesis of their collective feedback enhanced the app development. Unfortunately, most app development research does not include end users [23]. Our study extends the literature by suggesting that app development not only requires user input, but also that user input needs to be collected in diverse and iterative ways. No single modality is sufficient, and when diverse invested parties are involved, as is often the case in mental health services—especially when children or youth are involved—no single group of individuals can provide sufficient feedback. Our study describes the different ways and parties who were involved in the app development process, which can help others with models of how to apply human-centered design methods to digital mental health. Motivated by the ACTS model [20] and DDBT framework [21], this paper demonstrates the iterative nature of these methods as well as the importance of considering both the technological and service elements in the app. Indeed, in the development of any intervention designed to improve outcomes, it is important to include the intended community in the development of the intervention [31]. This study underscores the importance of human-centered design by showing that needed changes to the app were only identified with the inclusion of parties with similar characteristics of the end users (eg, parents of children with autism from low-resourced backgrounds) in the app development in various modalities.

Relatedly, to ensure that the needs and perspectives of diverse end users are met, it is crucial to involve individuals who are often overlooked by the research community and app developers. Our study focused on families of children with autism from low-resourced communities—a marginalized population that faces tremendous systemic barriers to accessing services [17]. As poverty is endemic to families of children with disabilities, it is necessary to elicit feedback from families to better understand ways to overcome fiscal barriers [32]. The need to include individuals from low-resourced backgrounds is especially true in relation to creating an app, as families with limited capital (eg, income) often face additional barriers to using technology [33].

Our study further extends the literature by showcasing ways to elicit feedback from families who are Spanish-speaking and low-resourced when developing an app. In the disability community, Spanish-speaking (vs English-speaking) families often face greater and unique obstacles to accessing services [34]. Thus, it is critical to develop interventions to improve their access to services. When considering a technological tool to improve access to services, the tool must be culturally responsive and demonstrate a “fit” with the intended population (eg, Spanish-speaking families) for the tool to be usable [35]. Unfortunately, most technological tools, including apps, are not culturally responsive [36]. To increase the cultural responsiveness of technological tools, it is important to garner input from individuals with different identities (eg, English and Spanish-speaking), acquire knowledge about different user experiences, and tailor features to the experiences of individuals [37]. Altogether, this study shares ways in which to elicit feedback from families who are often ignored both in research and app development.

Notably, there was a mismatch between the research goals and actual service use. For example, the app included an evaluation feature that was essential for collecting data and improving the app, but some participants reported the evaluative feature was less needed. The potential for conflicting perspectives between researchers and end-users is important to consider in moving forward with research about the app and in thinking about its sustainability. Regarding the former, moving forward, it may continue to be important to include the evaluative feature so that data can be collected to inform needed changes to the app. However, it may be equally important to consider the input from end users when thinking about the app’s features since data is dependent on usage. Ultimately, it is more important for the app to be useful in its deployment setting (vs a research setting) [23].

In considering the potential for differing needs between users and researchers, it may be appropriate to consider a longitudinal perspective. Instead of prioritizing the concerns of one group over the other, it may be possible to find a middle ground where the needs and interests of both parties are met. In the context of the evaluative feature of the app, this may mean retaining the evaluative feature during the development phase for data collection to determine its feasibility and effectiveness but removing the evaluative feature (or revisiting the need for it with end users) upon full deployment after the study ends.

Although unrelated to the technical features of the app, wraparound services were needed to support the use of the app. Specifically, some participants requested assistance from research team members to troubleshoot issues with the app and an additional training session demonstrating proper usage of the app’s functions. Such concerns are important to address, especially among families from low-resourced backgrounds who may possess limited digital literacy [33]. Thus, the presence of wraparound support may be tied to the successful usage of the app for some participants. The finding of needing additional wraparound services may be generalizable to other populations with limited digital literacy (eg, older adults). Again, this provides further demonstration of the importance of conceptualizing digital support not as products [20], but as technology-enabled services, and to focus design activities on both technological and service components [23].

Although an important launching point for developing an app for families of children with autism from low-resourced backgrounds, this study has a few limitations. The sample size was relatively small and, in some ways, homogenous. For example, all participants were from the same midwestern state and spoke either English or Spanish. Thus, there may be limited transferability of the findings to families from other states and individuals who speak other languages. Second, to date, we have only examined the development of the app. It remains to be seen whether the app can help promote service access and communication between the family and a navigator. Additional data would be helpful to determine whether the app can positively impact such child and family outcomes.

Our study suggests that it is feasible and worthwhile to include end users, especially end users who are often marginalized and not involved in research, in the development of interventions. This study demonstrates the many ways in which end users can provide feedback. Without unique opportunities for user involvement, issues with the app may have gone unaddressed. Researchers and developers may consider replicating our process to conduct human-centered design in their interventions.

## Appendix

Multimedia Appendix 1 Initial Identification of Features of the App.

Multimedia Appendix 2 The Initial Wireframe of the App.

Multimedia Appendix 3 Revised Wireframe.

Multimedia Appendix 4 Low-fidelity protoype of the app.

Multimedia Appendix 5 Minimally Viable Product.

Multimedia Appendix 6 List for Co-Designing Digital Supports with Community Partners.

Multimedia Appendix 7 Final version of the App.

## Abbreviations

ACTS: accelerated creation-to-sustainment
DDBT: discover, design, build, and test
PTI: Parent Training and Information Center

## Data Availability

The data sets generated and analyzed during this study are available in the National Database for Autism Research.
