# Supplementary material for: Leveraging Feedback From Families of Children With Autism to Create Digital Support for Service Navigation: Descriptive Study
Source: JMIR Form Res. 2024 Aug 14;8:e56043. doi: 10.2196/56043 (PMC11358655; doi:10.2196/56043)
Supplement: Multimedia Appendix 1 [file formative_v8i1e56043_app1.docx]

Multimedia Appendix 1: Initial Identification of Features of the App

*
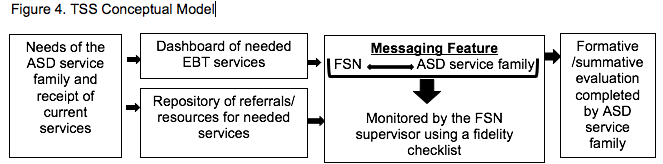
*

*Note:* ASD Service Family refers to the family of a young autistic child;

EBT refers to Evidence-Based Treatment; FSN refers to family service navigator
