# Supplementary figures and images for: Leveraging Feedback From Families of Children With Autism to Create Digital Support for Service Navigation: Descriptive Study
Source: JMIR Form Res. 2024 Aug 14;8:e56043. doi: 10.2196/56043 (PMC11358655; doi:10.2196/56043)

Multimedia Appendix 2: The Initial Wireframe of the App


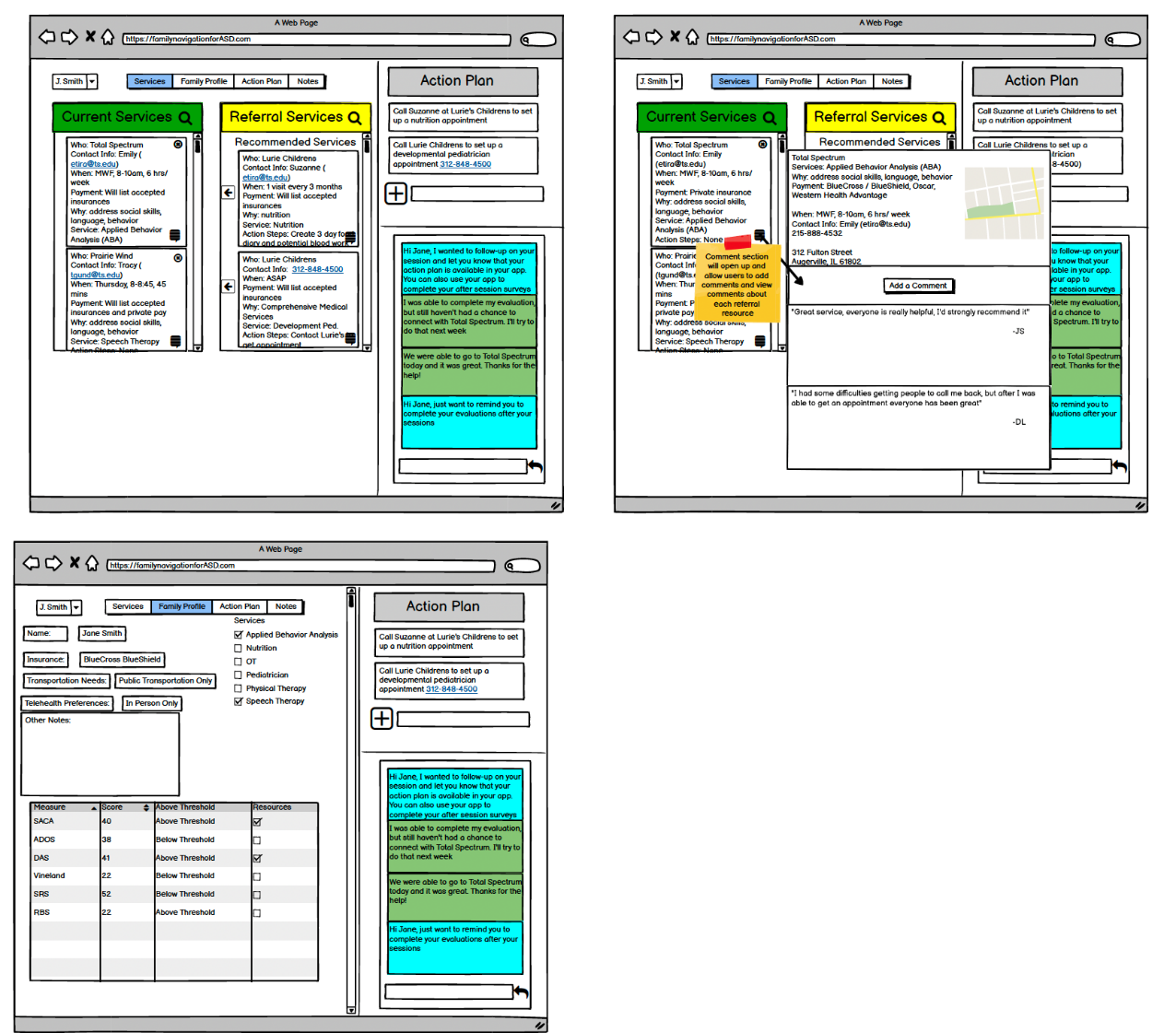

Supplement: Multimedia Appendix 2 [file formative_v8i1e56043_app2.docx]

Multimedia Appendix 3: Revised Wireframe

**
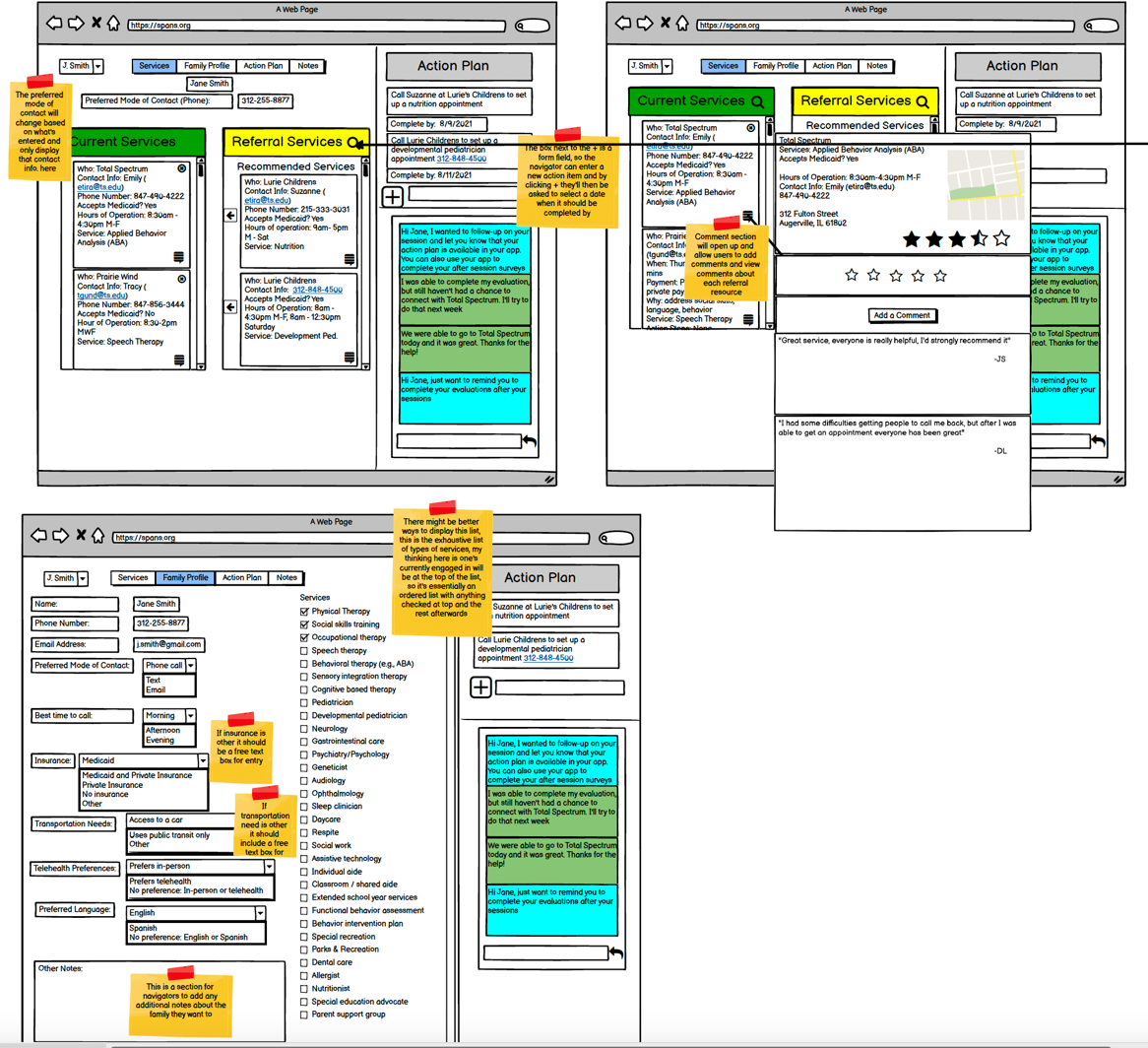
**

Supplement: Multimedia Appendix 3 [file formative_v8i1e56043_app3.docx]

Multimedia Appendix 4: Low-fidelity protoype of the app

**
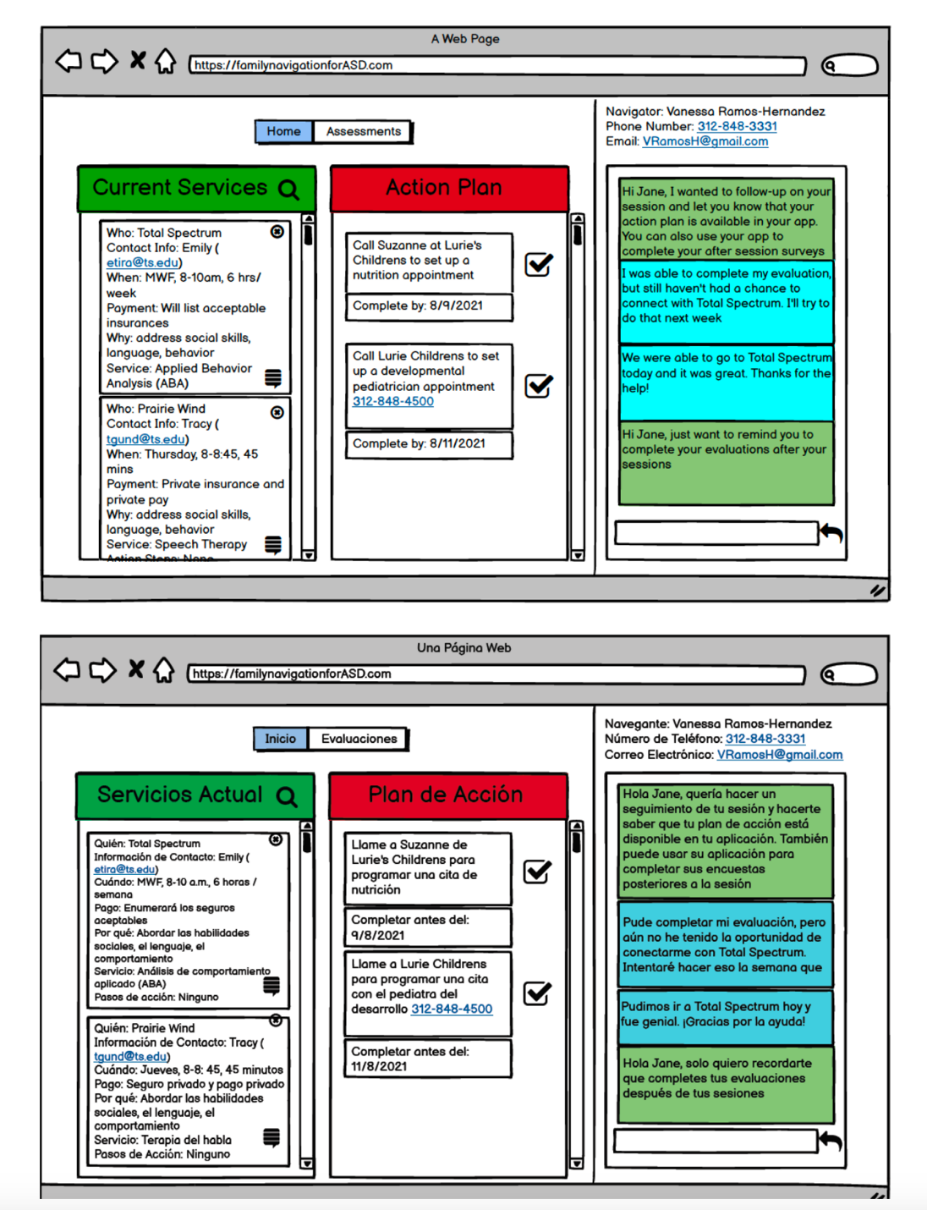
**

Supplement: Multimedia Appendix 4 [file formative_v8i1e56043_app4.docx]

Multimedia Appendix 5: Minimally Viable Product

**
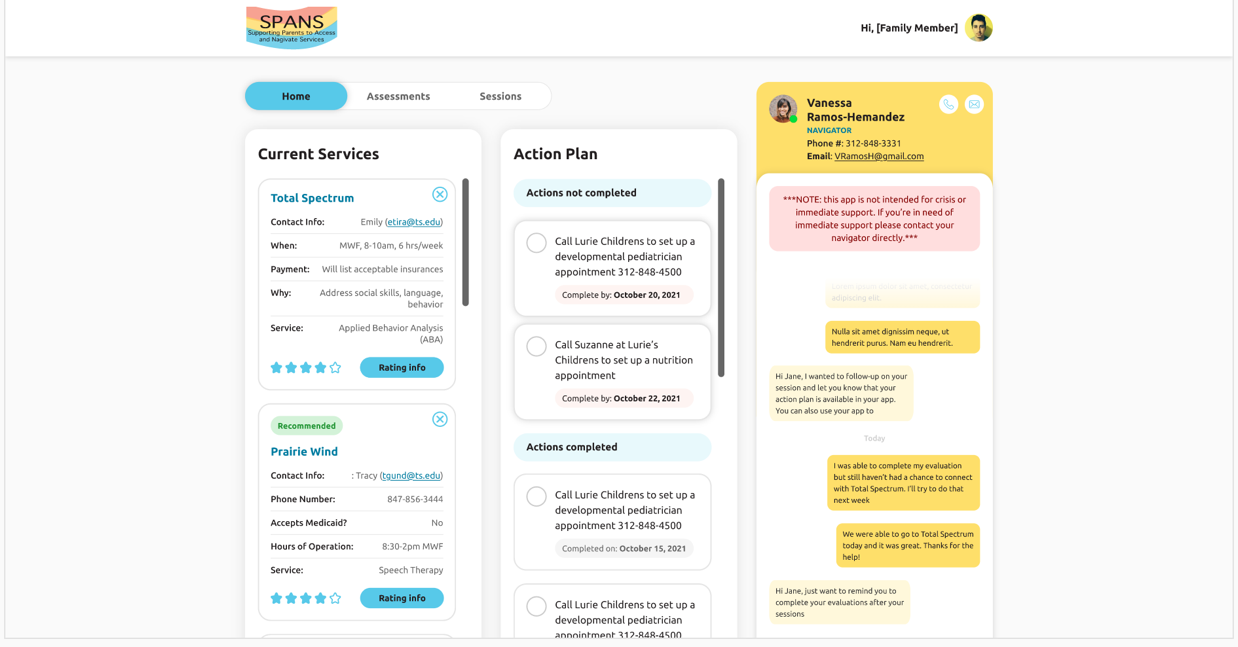
**

Supplement: Multimedia Appendix 5 [file formative_v8i1e56043_app5.docx]
