# Supplementary material for: Leveraging Feedback From Families of Children With Autism to Create Digital Support for Service Navigation: Descriptive Study
Source: JMIR Form Res. 2024 Aug 14;8:e56043. doi: 10.2196/56043 (PMC11358655; doi:10.2196/56043)
Supplement: Multimedia Appendix 6 [file formative_v8i1e56043_app6.docx]

Multimedia Appendix 6: List for Co-Designing Digital Supports with Community Partners

| Item | Steps to be taken |
| --- | --- |
| Identify relevant community organizations | -Talk to practitioners and intended users of the digital support to identify relevant community organizations  -Contact the community organizations to garner their interest in partnering  -Delineate the compensation for the community partner and their role (including tasks and responsibilities) in the partnership |
| Identify and consent individual users to partner in developing the digital support | -Operationally define the intended users of the digital support  -Talk to the community organization about the characterization of the intended users (i.e., who is an intended user?)  -Determine the compensation for individual users  -Consider and address accessibility and equity in identifying individual users  -With respect to accessibility: ensure recruitment materials reflect principles of universal design for learning, offer accommodations to individual users for their participation, ensure the digital support meets the accessibility guidelines per Section 508 of the American Rehabilitation Act  -With respect to diversity: hire cultural brokers and interpreters, make recruitment and data collection available in other languages, consider ways to meet the digital needs of families (e.g., offer smartphones with dataplans so families can access the digital support)  -Ask the community organization to help recruit intended users  -Consider other ways to recruit intended users  -With the community partner, decide on your needed sample size |
| Identify the methods for data collection | -Consider varying the modalities of data collection to include quantitative (e.g., surveys) and qualitative (e.g., interviews, focus groups, observations of meetings) data  -Create a timeline of waves of data collection (instead of collecting data at only one timepoint)  -Include methods of data collection that allow intended users to role play with the digital support |
