# Supplementary material for: Leveraging Feedback From Families of Children With Autism to Create Digital Support for Service Navigation: Descriptive Study
Source: JMIR Form Res. 2024 Aug 14;8:e56043. doi: 10.2196/56043 (PMC11358655; doi:10.2196/56043)
Supplement: Multimedia Appendix 7 [file formative_v8i1e56043_app7.docx]

Multimedia Appendix 7. Final version of the App


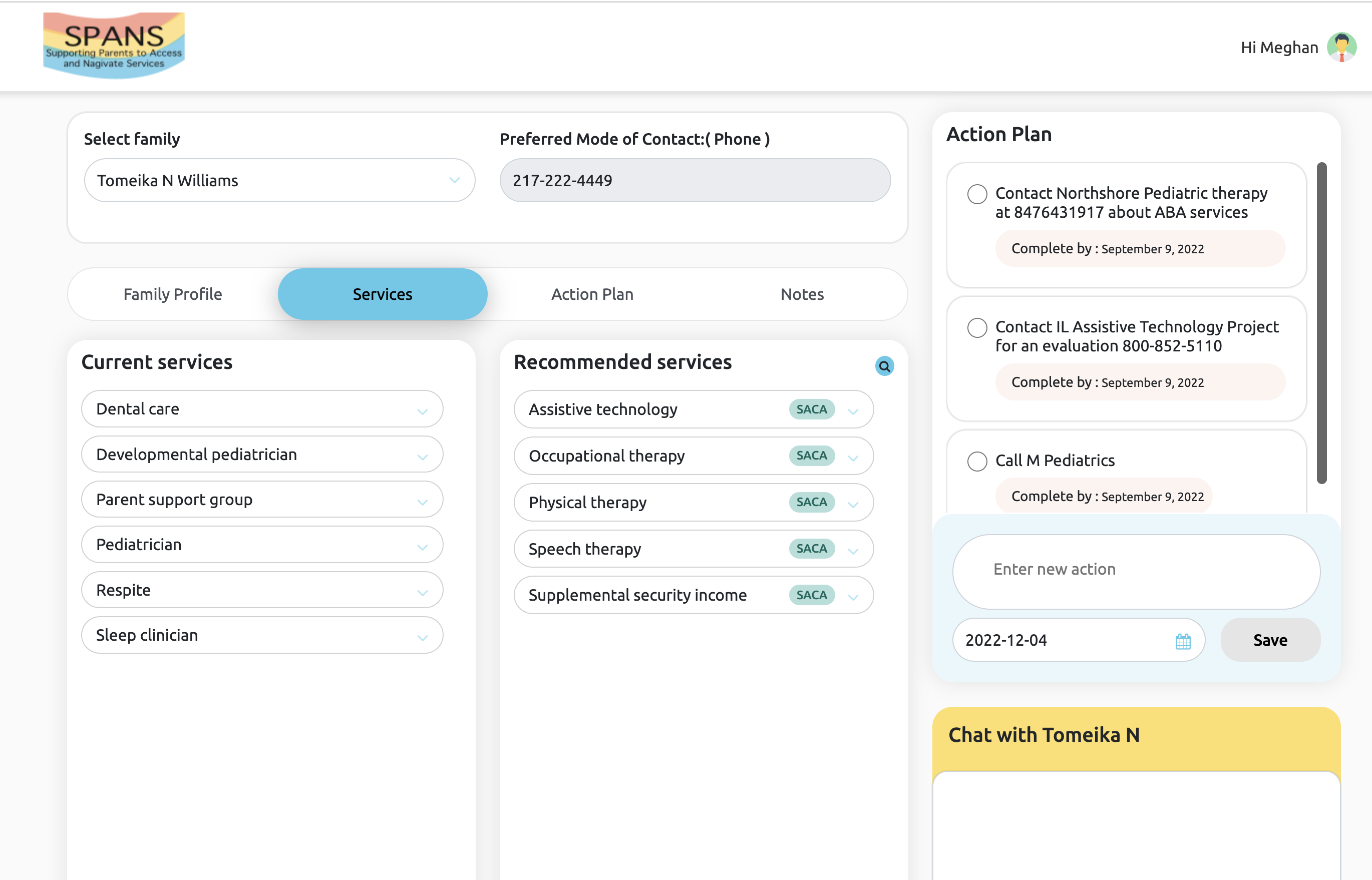


Figure 1. The profile of the family of a young autistic child


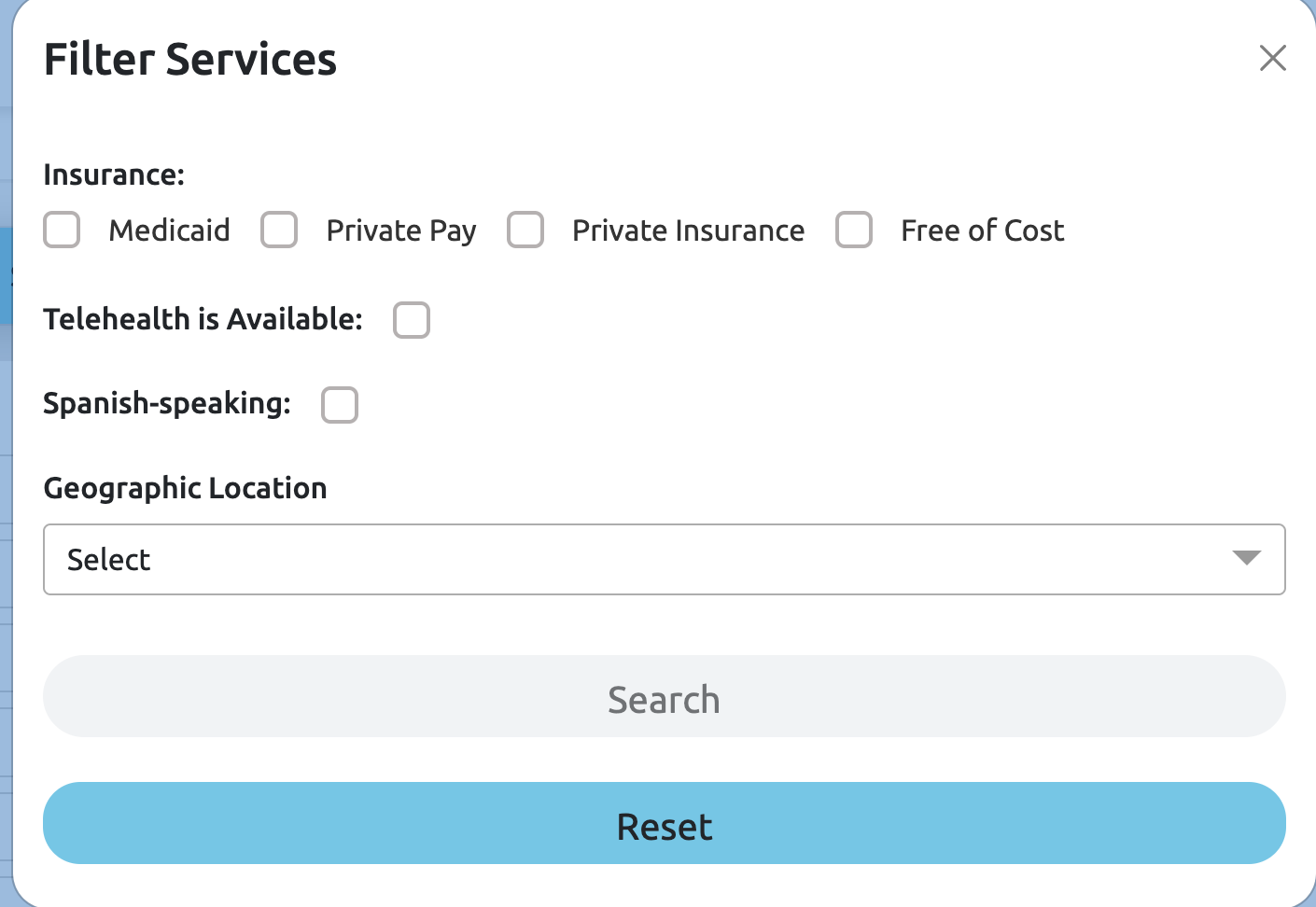


Figure 2. The filters that the navigator may select for a family to determine the parameters of the providers


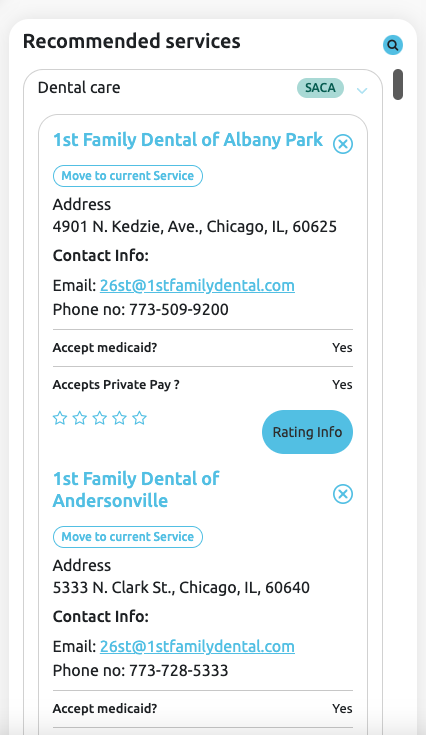


Figure 3. The profile of service providers that provide the recommended services for the family and meet the parameters set by the filters in Figure 2


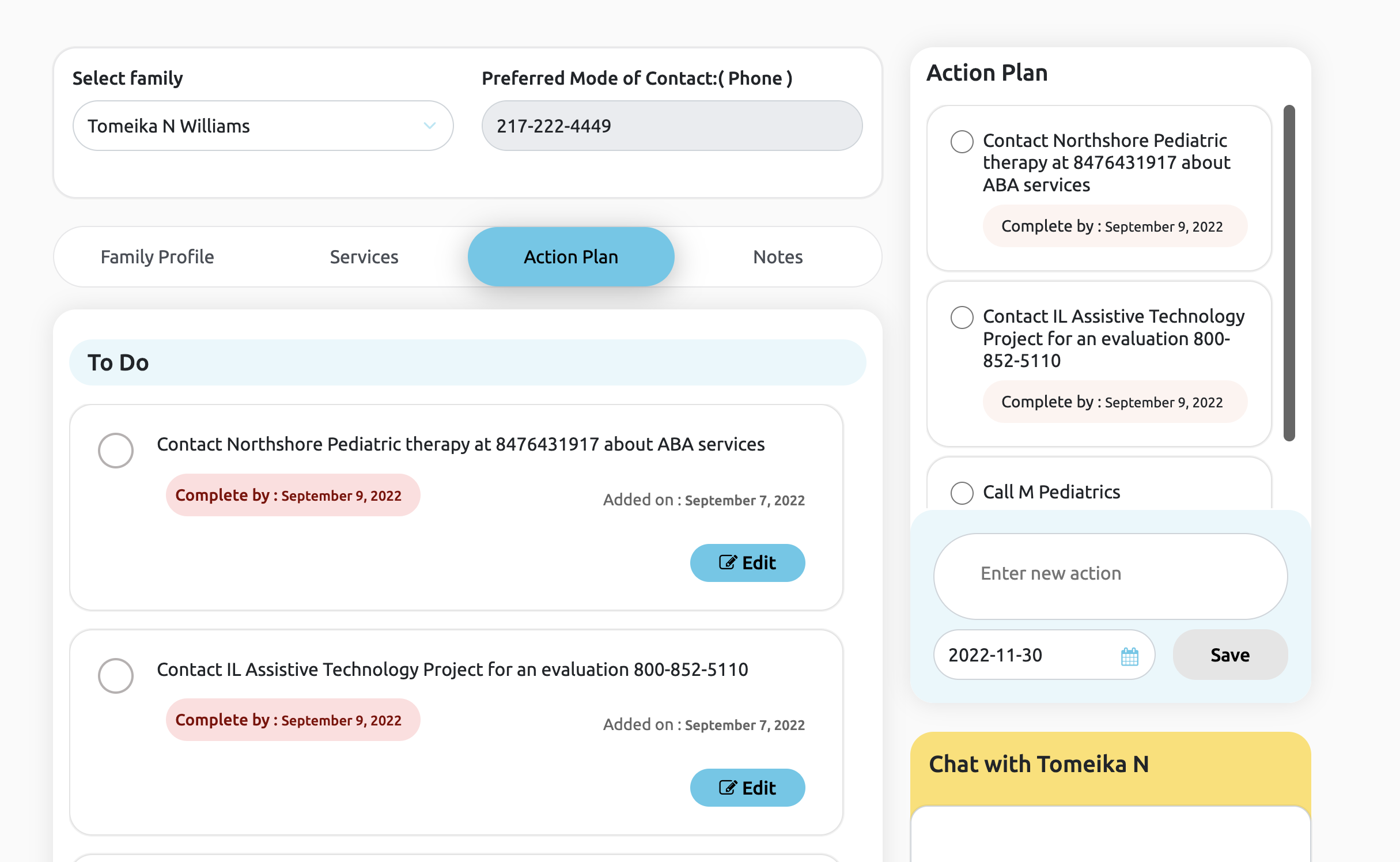


Figure 4. The action plan detailing the next steps for the family


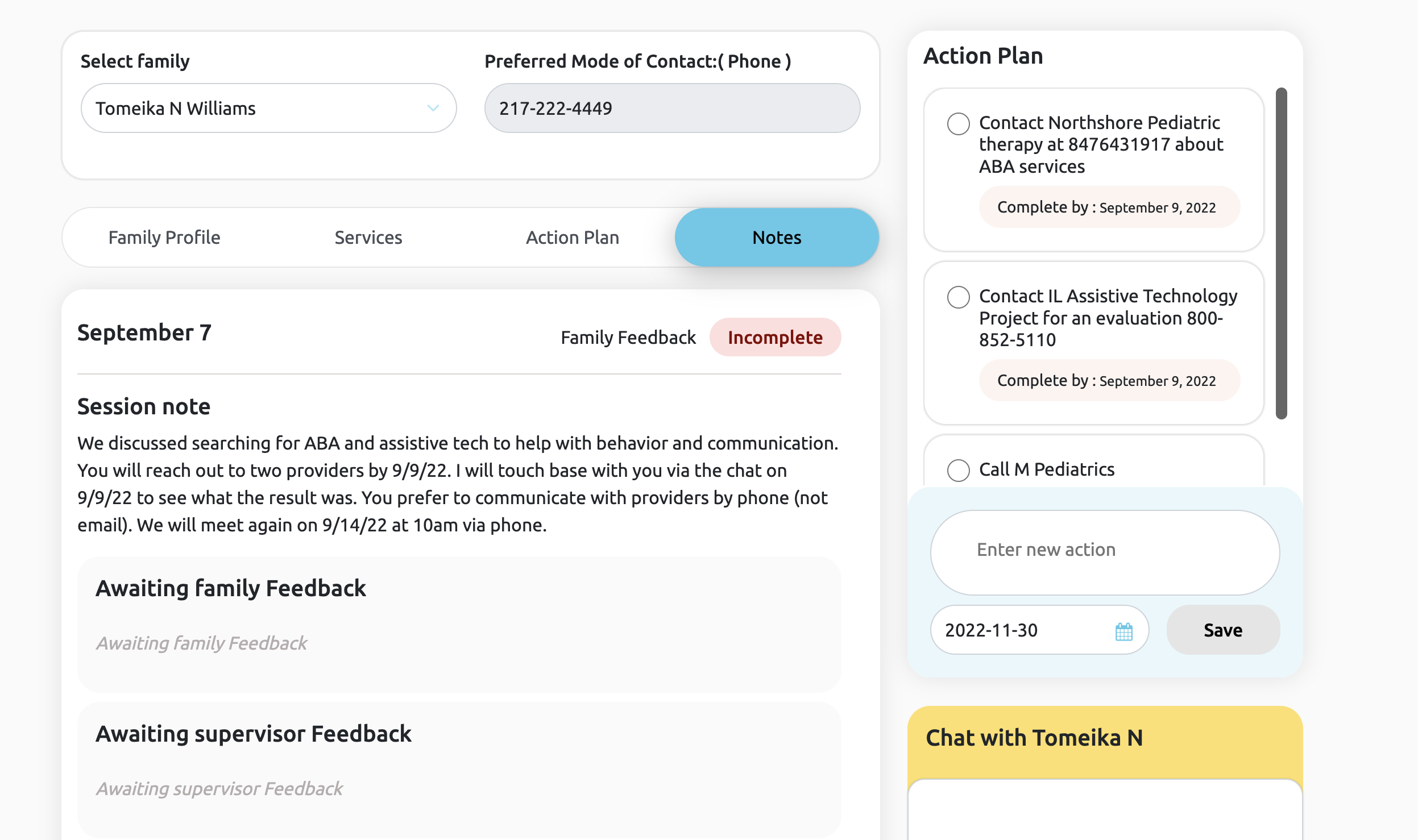


Figure 5. The session notes after each navigator meetings with the family


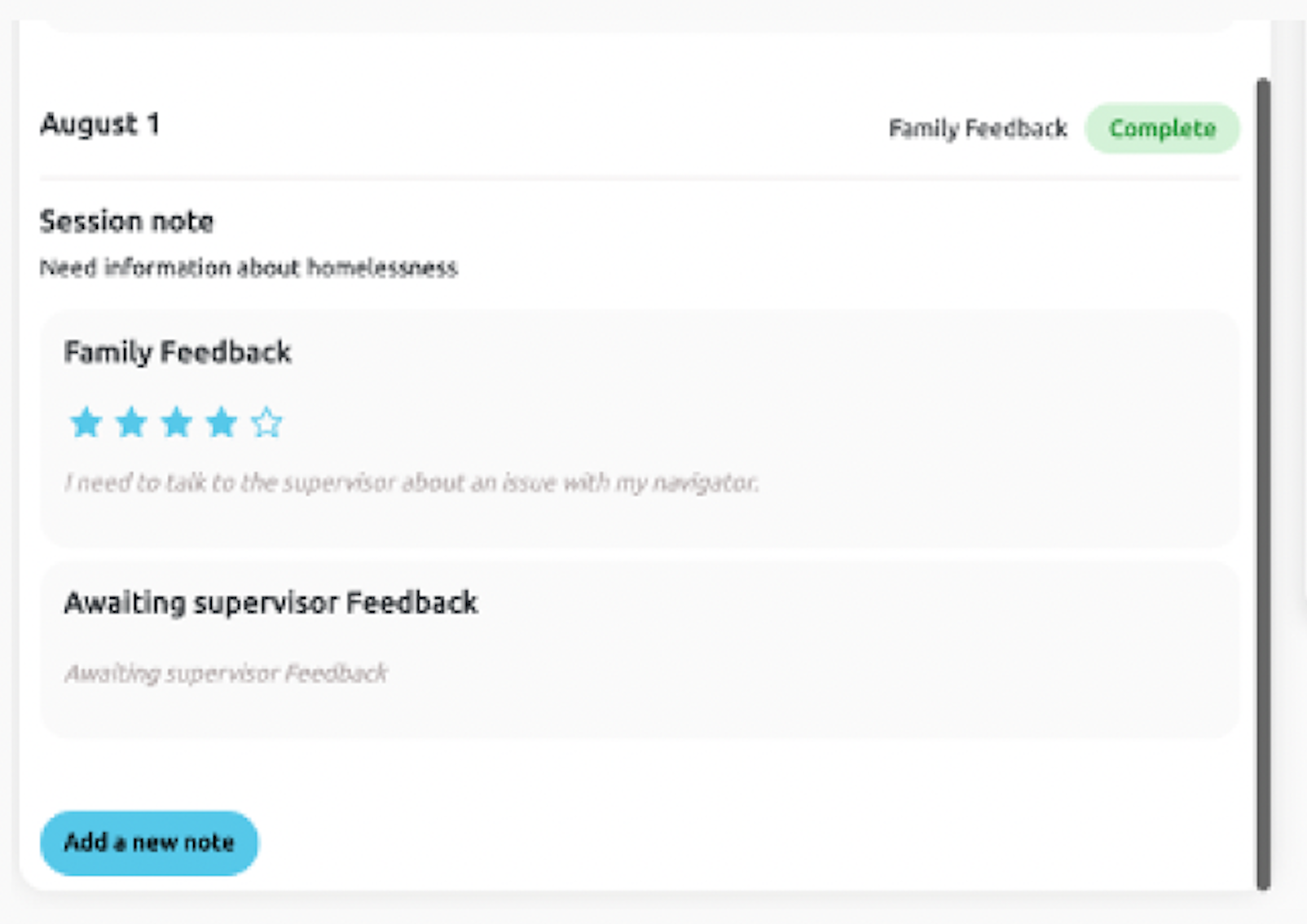


Figure 6. Evaluation of the navigator
